# Effective Treatment of Refractory Cluster Headache With Propranolol

**DOI:** 10.7759/cureus.70391

**Published:** 2024-09-28

**Authors:** Rami Algahtani, Khalid Basamih, Abdullah Aldawsari, Abdulaziz Alyazidi, Yazan Baqadir

**Affiliations:** 1 Department of Medicine and Surgery, College of Medicine, Umm Al-Qura University, Makkah, SAU

**Keywords:** beta blocker, cluster headache, propranolol, refractory, relieve

## Abstract

Cluster headache (CH) is a debilitating neurological condition characterized by excruciating unilateral pain, often accompanied by autonomic symptoms. We present a compelling case report detailing the experience of a 22-year-old male who suffered from refractory CH and exhibited a remarkable response to propranolol. This unique case highlights the potential effectiveness of propranolol in alleviating CH symptoms that are resistant to conventional therapies. Moreover, the absence of reported side effects on such a low dose further underscores its appeal as a treatment option. This case report sheds light on a potential avenue for managing refractory CH, offering hope for individuals who struggle with this debilitating condition.

## Introduction

Headache is one of the most common neurological complaints in Saudi Arabia [1]. Cluster headache (CH) is described as severe, strictly unilateral pain attacks that are orbital, supraorbital, temporal, or any combination. Each episode of CH lasts 15-180 minutes and occurs once every other day to eight times a day [2]. Typical CH pain may be associated with ipsilateral conjunctival injection, lacrimation, nasal congestion, rhinorrhea, forehead and facial sweating, miosis, ptosis, eyelid edema, restlessness, or agitation [3]. The pathophysiological process of CH is not entirely understood. However, there are risk factors that increase the possibility of this pain, such as male gender, age of more than 30, consumption of alcohol, prior brain surgery or trauma, and family history [2].

Multiple oral therapeutic options are available, including verapamil, topiramate, and lithium, and have shown successful results in alleviating the pain and/or frequency of CH attacks. Little is known about how to manage refractory cases. Given the rarity of the disease, the role of beta blockers remains controversial, with no updated literature or evidence in this field [4].

This case report presents a 22-year-old male with CH refractory to standard treatment and responded to beta blockers.

## Case presentation

A 22-year-old male presented to the neurological clinic complaining of a left periorbital severe paroxysmal headache. These headaches were described as extremely painful, occurring two to three times a day, usually between 9 am and 4 pm, and lasting for 45 minutes with no radiation to other sites, aggravated by noise and bright lights, with left conjunctival hyperemia as well as an increase in lacrimation, nausea, and vomiting. There was no rhinorrhea, neck stiffness, fever, fatigue, weight loss, and night sweating. There were also no reported visual symptoms. His past medical history was unremarkable but included otolaryngologist and ophthalmologist visits, which revealed normal exams.

**Figure 1:**
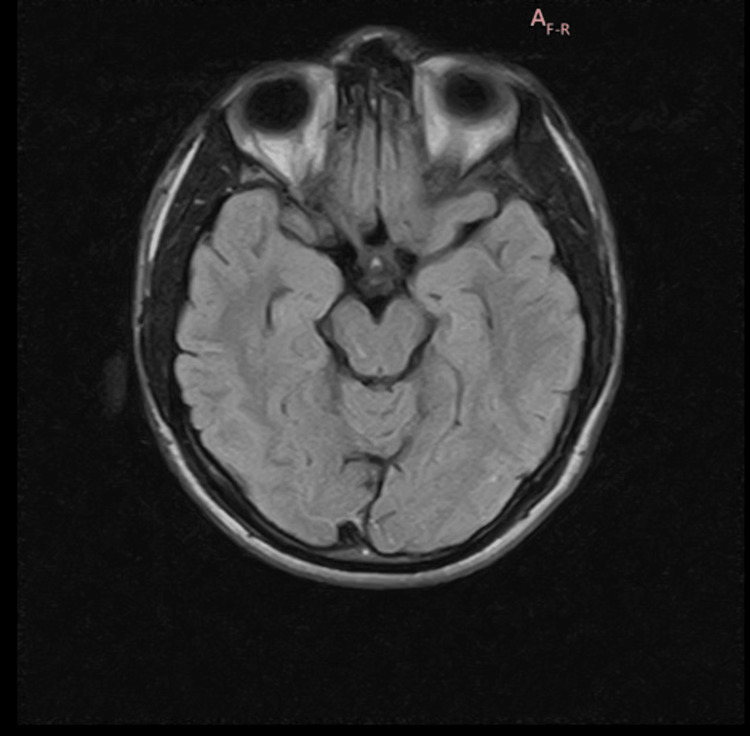
T2 axial FLAIR sequence of MRI brain showing no abnormalities

**Figure 2:**
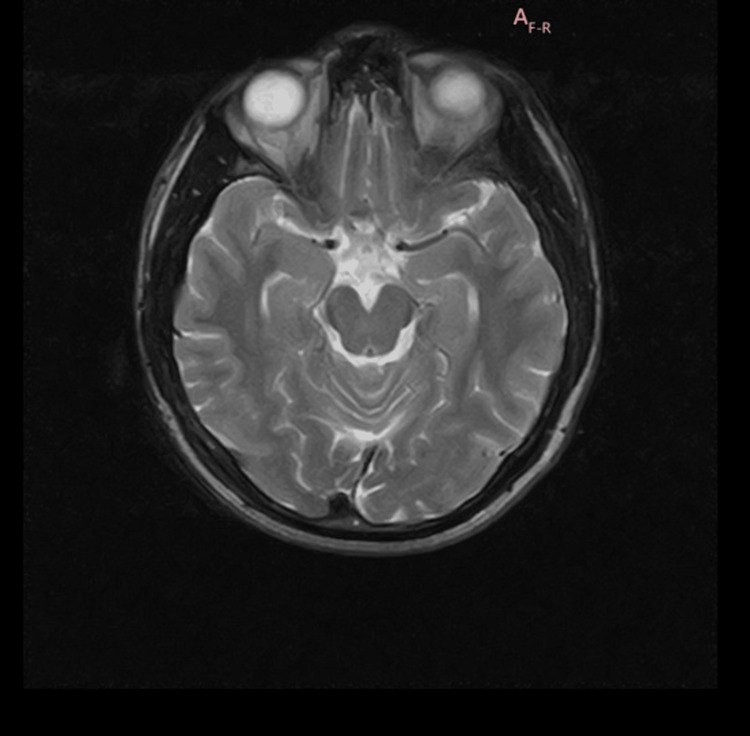
T2 sequence of MRI brain without contrast showing no abnormalities

Over time, the headache worsened and led the patient to visit the emergency room (ER) twice; the first time, he presented with a severe headache associated with conjunctival erythema and lacrimation in the left eye, nausea, and vomiting. The patient was treated with IV paracetamol at that time, and blood tests did not show significant results. The second ER visit was similar to the first and treated with IV paracetamol. After visiting the neurology clinic, the patient was diagnosed with CH and started on calcium channel blockers, verapamil 40mg two times a day, and eletriptan 40mg as an abortive drug because home oxygen tanks were not available. Magnetic resonance imaging (MRI) of the brain was done, and no abnormalities were shown (Figures 1-2).

After a month, the patient returned for follow-up and reported no improvement despite maximizing the dose of verapamil to 240 mg/day. Topiramate was added on an escalating dose to target 50mg twice daily. Initially, the patient's headache intensity decreased from a 10 to a 4 on the pain scale, though the frequency remained the same. He underwent bilateral occipital nerve block with triamcinolone and lidocaine with an excellent response in terms of headache frequency and severity. After one month, the headache returned aggressively despite a high dose of verapamil and topiramate. Another trial of occipital nerve injection resulted in temporary improvement for three weeks, but the headaches recurred afterward. The decision was made to add beta blocker propranolol 10mg twice daily after tapering off verapamil, which the patient had the least benefits from.

On the next visit, after a month, the patient reported great relief after adding propranolol. There were no further headache episodes. Topiramate was stopped, and he maintained his improvement solely on beta blockers.

## Discussion

This case involves a 22-year-old man with a nine-year history of severe paroxysmal periorbital headaches, accompanied by conjunctival hyperemia, lacrimation, nausea, and vomiting in the left eye, which met the International Headache Society criteria for CH. His condition was refractory to standard preventive treatments, including calcium channel blockers, topiramate, and occipital nerve injections, but showed sustained improvement with propranolol.

The clinical use of calcium channel blockers in neurological diseases focuses on two main therapeutic fields: (1) preventing delayed cerebral ischemia after subarachnoid hemorrhage with nimodipine, (2) and migraine prophylaxis with verapamil as an effective option [5]. Verapamil is the first-line preventive treatment for CH [3]. A study in 2009 reported the mechanism of verapamil; it is an L-type calcium channel blocker, as well as a blocker of other calcium channels (T-, P-, and possibly N- and Q-type Ca (2+) channels). Currently, no specific mechanism of action of verapamil for the treatment of CH can be identified [5]. A study in 2004 reported that verapamil was given to 18 patients with chronic CH and 52 patients with episodic CH during cluster periods, and complete headache relief was achieved in 49 (94%) of 52 episodic and 10 (55%) of 18 chronic CH; the largest required dosage was 200-480 mg [6]. The brain stem and trigeminal complex are both covered in serotoninergic receptors. The triptan therapeutic action is hypothesized to be caused by antagonizing these receptors. This impact could be achieved by inhibition of trigeminal nerve endings in large cerebral vessels, direct vasoconstriction of these associated vessels, and neuronal inhibition of more central hypothalamic lesions [7]. A study in 2007 reported that triptans were utilized by a total of 180 CH patients, with 71.1% reporting successful outcomes [8]. In this case report, our patient used verapamil up to 240mg daily and eletriptan as needed, with no improvement in severity or frequency in the paroxysmal attacks.

Topiramate treatment was associated with rapid improvement in 10 CH patients. In addition, cluster remission occurred in one to three weeks in nine patients, and the cluster period duration was reduced. The mechanism for this response may be related to gamma-aminobutyric acid (GABA) augmentation [9]. The second choice is topiramate if verapamil and lithium are ineffective, contraindicated, or discontinued because of side effects. In addition, the dosage should be titrated slowly to manage side effects since they are frequent and include paraesthesia, mood swings, and speech disturbances [10]. Topiramate partially benefited our patients regarding the severity of the attacks, but the frequency remained the same, with a substantial effect on quality of life.

The mechanisms of greater occipital nerve (GON) blockade efficacy in CH are unknown. However, in neurophysiological research, Busch demonstrated functional connectivity between the trigeminal sensory afferents and the cervical pathway in healthy subjects [11]. Therefore, authors hypothesize that GON blockade in CH centrally desensitizes the central nervous system area that overlaps the trigeminal and cervical sensory afferent projections, possibly altering the trigeminal autonomic response pathway [12]. GON block has been indicated for several common types of headaches, including CH, classic migraine, occipital neuralgia, and cervicogenic headaches. Therefore, it is a second-line treatment when other methods have failed [11] in patients who have contraindications to using triptans or who report sumatriptan side effects [12]. A case report in 2006 discussed the outcome of GON blockage in a patient with CH and showed that the clinical response was better. He was pain-free in a matter of minutes, and following the injection, the patient experienced no side effects. After one month, he noted significant improvement in both frequency and intensity. Then, after one year of sitting, he was still pain-free [12]. In our patient, the headache stopped for two weeks after the GON blockage was done. After some time, the headache returned, but this time it was more severe. The procedure was repeated but showed temporary benefits.

Propranolol is a non-selective beta blocker that can freely cross the blood-brain barrier (BBB) to modulate its effect because it is highly lipid soluble. The effectiveness of propranolol in preventing migraines has been proven in several studies [13]. To the best of our knowledge, there are no recent studies discussing the outcome of propranolol or other beta blockers in patients with CH. A study in 1972 reported the effectiveness of propranolol in treating the symptoms of CH, assuming that it resulted from the effect of the drug on histamine activity inhibition [4]. Another study in 1980 mentioned that another beta blocker, timolol, was effective in relieving and decreasing CH [14].

In this case, propranolol had a considerable impact on alleviating patient symptoms, with no reported side effects at such a low dose. Moreover, a prolonged remission period reduces the severity of CH, improving the patient's quality of life. 

It is planned for the patient to stay on propranolol as a prophylactic measure for at least three months while tapering off other medications. Further large-scale research is required to determine the efficacy and safety of this therapeutic strategy, particularly in comparison to other standard therapeutic options.

## Conclusions

CH is a severe form of primary headache that may profoundly impact the quality of life of affected individuals. Although the current treatment strategies are effective, refractory cases may benefit from using beta blockers, even in low doses, either as a single agent or an adjunct.
